# Trauma/hemorrhagic shock instigates aberrant metabolic flux through glycolytic pathways, as revealed by preliminary ^13^C-glucose labeling metabolomics

**DOI:** 10.1186/s12967-015-0612-z

**Published:** 2015-08-05

**Authors:** Angelo D’Alessandro, Annie L Slaughter, Erik D Peltz, Ernest E Moore, Christopher C Silliman, Matthew Wither, Travis Nemkov, Anthony W Bacon, Miguel Fragoso, Anirban Banerjee, Kirk C Hansen

**Affiliations:** Department of Biochemistry and Molecular Genetics, University of Colorado Health Sciences Center, East 17th Ave, Aurora, CO 12801 USA; Department of Surgery, University of Colorado, Aurora, CO USA; Denver Health Medical Center, Denver, CO USA; Bonfils Blood Center, Denver, CO USA

**Keywords:** Lactate, Acidosis, Succinate, Gluconeogenesis, Glutaminolysis, Mass spectrometry

## Abstract

**Background:**

Metabolic derangement is a key hallmark of major traumatic injury. The recent introduction of mass spectrometry-based metabolomics technologies in the field of trauma shed new light on metabolic aberrations in plasma that are triggered by trauma and hemorrhagic shock. Alteration in metabolites associated with catabolism, acidosis and hyperglycemia have been identified. However, the mechanisms underlying fluxes driving such metabolic adaptations remain elusive.

**Methods:**

A bolus of U-^13^C-glucose was injected in Sprague–Dawley rats at different time points. Plasma extracts were analyzed via ultra-high performance liquid chromatography-mass spectrometry to detect quantitative fluctuations in metabolite levels as well as to trace the distribution of heavy labeled carbon isotopologues.

**Results:**

Rats experiencing trauma did not show major plasma metabolic aberrations. However, trauma/hemorrhagic shock triggered severe metabolic derangement, resulting in increased glucose levels, lactate and carboxylic acid accumulation. Isotopologue distributions in late Krebs cycle metabolites (especially succinate) suggested a blockade at complex I and II of the electron transport chain, likely due to mitochondrial uncoupling. Urate increased after trauma and hemorrhage. Increased levels of unlabeled mannitol and citramalate, metabolites of potential bacterial origin, were also observed in trauma/hemorrhagic shock rats, but not trauma alone or controls.

**Conclusions:**

These preliminary results are consistent with observations we have recently obtained in humans, and expand upon our early results on rodent models of trauma and hemorrhagic shock by providing the kinetics of glucose fluxes after trauma and hemorrhage. Despite the preliminary nature of this study, owing to the limited number of biological replicates, results highlight a role for shock, rather than trauma alone, in eliciting systemic metabolic aberrations. This study provides the foundation for tracing experiments in rat models of trauma. The goal is to improve our understanding of substrate specific metabolic derangements in trauma/hemorrhagic shock, so as to design resuscitative strategies tailored toward metabolic alterations and the severity of trauma.

**Electronic supplementary material:**

The online version of this article (doi:10.1186/s12967-015-0612-z) contains supplementary material, which is available to authorized users.

## Background

Severe metabolic deregulation is an established hallmark of major traumatic injury [1]. Further, metabolic aberrations are key instigators of the clinical sequelae of trauma and hemorrhagic shock (T/HS), including inflammation, activation of the complement system, and coagulopathy [2, 3]. Conventional measures of biochemical imbalance following T/HS, specifically based deficit and acidosis, have been shown to correlate with patient outcome [4, 5]. As such, clinical endpoints for resuscitation have historically included plasma lactate levels, the anion gap and base deficit [4, 5]. However the assessment of these parameters alone represents an inaccurate [6] or insufficient [7] descriptor of the metabolic endpoints of major injury. The predictive value of these conventional assessments may be confounded by hypoalbuminemia, elevated PaCO_2_, and unmeasured anions [6]. It has been proposed that the strong ion gap (SIG) more accurately accounts for these unmeasured anions, but even this physiochemical approach is limited in its scope by failing to consider the contribution of the majority of biochemical processes occurring simultaneously in the injured patient. Instead, improved understanding of the post-injury metabolome could facilitate more appropriately targeted resuscitation strategies.

Catabolism, acidosis, and insulin resistance with resultant hyperglycemia (“traumatic diabetes”) are examples of defined metabolic phenotypes (metabotypes) contributing to secondary injury following T/HS [8–14]. However, the complex biochemical constitutions and interactions of such metabotypes remain to be elucidated. Recent advances in omics disciplines are providing clinicians with unprecedented tools to better describe metabolic underpinnings [9]. Metabolomics, the comprehensive study of small molecules (molecular weight <1.5–2 kDa), has employed mass spectrometry (MS)-based investigations with improved sensitivity and specificity in metabolic coverage [14, 15]. MS-metabolomics can also be used to trace the specific flux of substrates through metabolic pathways using stably labeled compounds (e.g. uniformly labeled U-^13^C-glucose) [16]. In so doing, we can gather information about dynamic metabolic fluxes through given pathways (e.g. glycolysis and Krebs cycle), other than monitoring the steady-state levels of specific metabolites. Results from these flux analyses deliver mechanistic insights, and thus indicate potential targets for tailored resuscitative strategies, similar to what has recently been proposed for ischemic/reperfusion injury [15].

We recently reported on a plasma MS-metabolomic analysis from a small cohort of severely injured human trauma patients [14]. Results were consistent with existing knowledge about the role of T/HS in the promotion of catabolic, acidotic and hyperglycemic metabotypes, but delivered improved specificity and broader coverage of pathways at steady state conditions. Increased levels of glycolytic, Krebs cycle, proteolytic and lipolytic/fatty acid metabolites demonstrated a hyper-catabolic state, with anticipated consequences on acid/base balance and glucose utilization [14]. Indeed, mobilized amino acids with acidic pKas demonstrate anionic biochemical behavior at physiologic pH. Also, increased plasma levels of di- or tri-carboxylic acids have potential for significant contribution to non-lactate acidosis [14]. However, where and how the cell uses these substrates or accumulates these products following trauma or hemorrhagic shock is not explicitly clear. For example, we observed markers of glutaminolysis and proteolysis (amino acid accumulation), providing possible alternative carbon source than glucose to drive increased levels of Krebs cycle intermediates [14, 17]. However, in the absence of flux analysis from heavy labeled glucose, we could not rule out the possibility that glucose catabolism might provide the main carbon source to fuel Krebs cycle anaplerosis. Conversely, accumulating Krebs intermediates could be consistent with decreased oxidative phosphorylation at the mitochondria resulting from electron transport chain uncoupling [15]. We also documented the accumulation of hypoxanthine and urate in a rat model [18], presumably from purine catabolism. Urate, a potent anti-oxidant, is theorized to play a role in adaptive responses to preserve post-shock redox poise [14, 15]; thus clear understanding of upstream biochemical contributions would be advantageous in designing targeted resuscitation strategies to prevent secondary injury. In order to determine the most significant factors influencing post-injury metabotypes, targeted labeling experiments are required to elucidate the specific dynamics of substrate flux across the pathways in different stages (e.g. early or late trauma, either alone or in combination with hemorrhage).

Controlled animal models are necessary to define precise metabolic consequences following trauma and hemorrhagic shock in isolation and in concert. These models afford a degree of experimental control (i.e. decreased biological variability and controlled severity of trauma and shock) that is not possible in the human trauma population. In this preliminary study we exploit heavy carbon tracing from uniformly labeled glucose, and mass spectrometry-determined isotopologues to investigate substrate flux in glycolysis and Krebs cycle pathways following trauma or hemorrhagic shock in the rat. We hypothesize that trauma and hemorrhagic shock will evoke differential metabolic changes and that major contributions to these pathways will be non-glucose under hemorrhagic shock conditions.

## Methods

### Animal model

Animal experiments were performed under a protocol approved by the Institutional Animal Care and Use Committee at the University of Colorado Denver. All animals were maintained in the accordance with the recommendations of the *Guide for the Care and Use of Laboratory Animals*. Animals were housed under barrier-sustained conditions with 12-h light–dark cycles and allowed free access to food and water before use.

Sprague–Dawley rats (n = 8) weighing 350–500 mg (Harlan Labs, Indianapolis, IN, USA) were anesthetized with 50 mg/kg Pentobarbital sodium via intraperitoneal injection. A tracheostomy was performed. The femoral artery and vein were then cannulated and mean arterial pressure (MAP) was monitored using a ProPaq invasive monitoring device. Rectal temperature assured euthermia. Blood was withdrawn from the femoral artery at a baseline time point, followed by an intravenous injection of labeled carbon glucose (iLC) (U-^13^C_1-6_-glucose—no. 389374, Sigma-Aldrich Corp., St. Louis, MO, USA). This “bolus” was defined as 5 ml/Kg at a rate of 3 cc/min (~2.15 cc in 40 s) of D5NS solution (5% labeled dextrose in normal saline), consistent with similar experiments in the literature [19]. Blood draws of 0.5 mL in heparinized tubes were performed at 5, 10, 15 and 35 min from iLC. Blood samples are centrifuged at 1,000 RCF for 15 min at 4°C. Plasma was removed and centrifuged again at 12,500 RCF for 6 min at 4°C. Samples were flash frozen and stored at −80°C prior to batch metabolomics analyses.

**Fig. 1:**
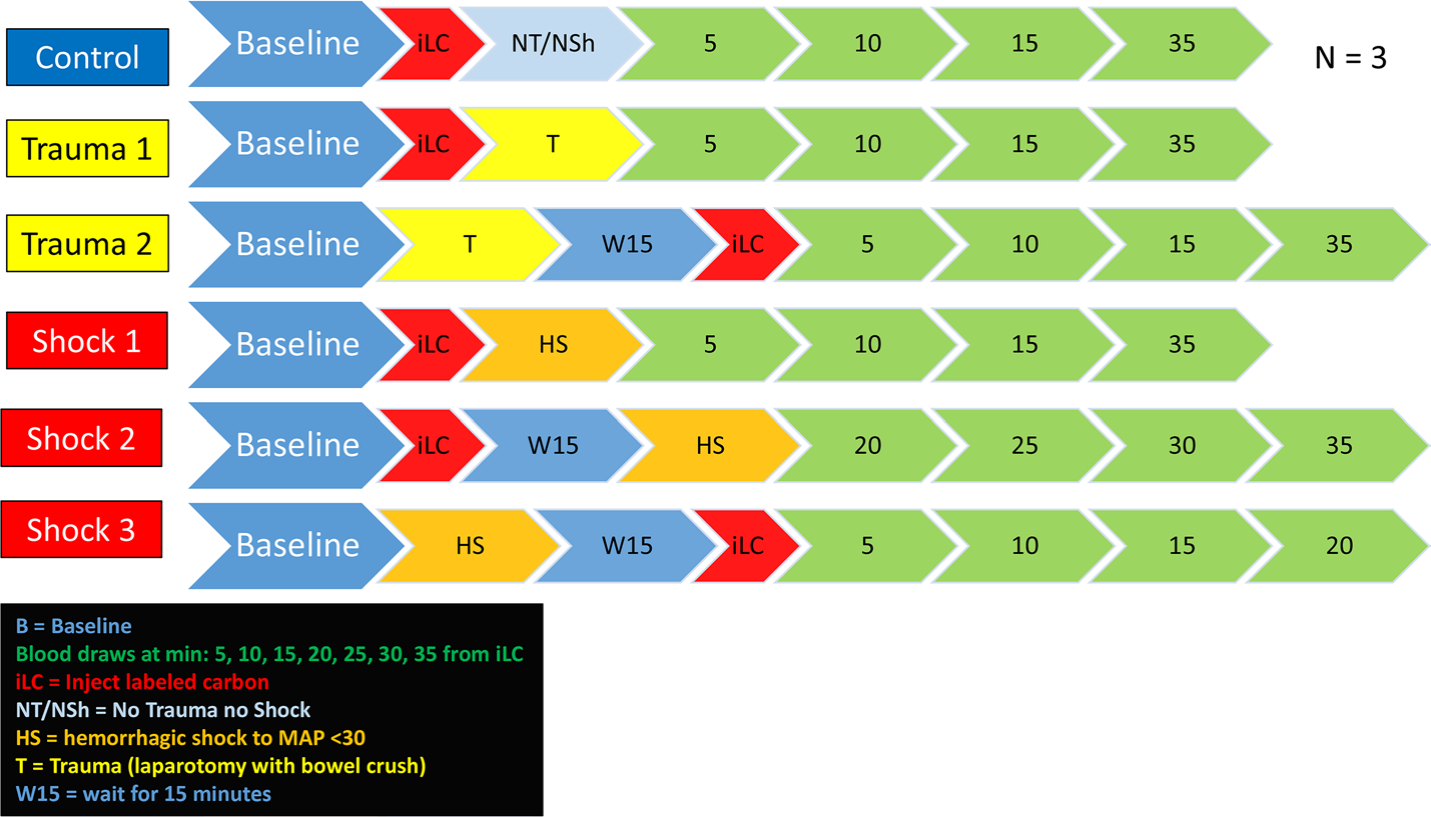
A layout of the experimental model adopted in this study is shown. Injections of labeled ^13^C-glucose (iLC) were performed after *baseline* (B) blood withdrawal in controls (no trauma/hemorrhagic shock—NT/NSh, n = 3), trauma (T *yellow*) or trauma/hemorrhagic shock rats (HS *orange*). Blood was thus collected and plasma separated at different time points (minutes from iLC are indicated in the *green arrows*).

Three rats were used as controls (no trauma or hemorrhagic shock). “Trauma” rats (n = 2) underwent 3 cm midline laparotomy and bowel crush. Trauma rats received iLC prior to laparotomy (T1) or following laparotomy (T2). “Shock” rats (n = 3) underwent controlled hemorrhage to MAP <30 mmHg for 35 min. Shock rats received iLC immediately before (HS1), 15 min before hemorrhage (HS2) or 15 min into hemorrhage (HS3). All rat models are detailed in Fig. 1 [20].

### Metabolomics analyses

Plasma samples (10 µl) were extracted in ice-cold lysis/extraction buffer (methanol:acetonitrile:water 5:3:2) at 1:25 dilutions. Technical variability and sample handling were controlled for by spiking in heavy labeled ^13^C_6_-lysine and ^13^C_6_-arginine (10 µM), and the xenometabolite 5-fluorouracil (25 µM) in the lysis buffer, as previously reported [21].

Samples were then agitated at 4°C for 30 min and centrifuged at 10,000*g* for 15 min at 4°C. Protein and lipid pellets were discarded, while supernatants were used for metabolomics analyses.

Metabolomics analyses were performed as previously reported [18, 22]. Ten µl of sample extracts were injected onto an UPLC system (Ultimate 3000, Thermo, San Jose, CA, USA) and run on a Kinetex XB-C18 column (150 × 2.1 mm, 1.7 µm particle size—Phenomenex, Torrance, CA, USA) at 250 µl/min (mobile phase: 5% acetonitrile, 95% 18 mΩ H_2_O, 0.1% formic acid—3 min isocratic run). The UPLC system was coupled online with a QExactive system (Thermo, San Jose, CA, USA), scanning in Full MS mode (2 µscans) at 70,000 resolution in the 60-900 m/z range, 4 kV spray voltage, 15 sheath gas and 5 auxiliary gas, operated in negative and then positive ion mode (separate runs). Calibration was performed before each analysis against positive or negative ion mode calibration mixes (Piercenet—Thermo Fisher, Rockford, IL, USA) to ensure sub ppm error on the intact mass. Metabolite assignments were performed using the software Maven (Princeton, NJ, USA), upon conversion of .raw files into.mzXML format through MassMatrix (Cleveland, OH, USA). The software allows for peak picking, feature detection and metabolite assignment against the KEGG pathway database. Assignments were further confirmed against chemical formula determination (as gleaned from isotopic patterns and accurate intact mass), isotopologue distributions (corrected for natural abundance) in presence of ^13^C labeling from heavy U-^13^C_1,2,3,4,5,6_-glucose and retention times against a library of 619 standard compounds (SIGMA Aldrich, St. Louis, MO, USA; MLSMS, IROATech, Bolton, MA, USA).

Integrated peak area values for each metabolite (including the isotopologue distributions) were exported into.csv files and results were graphed through GraphPad Prism 5.0 (GraphPad Software Inc., La Jolla, CA, USA). Figure panels were assembled through Photoshop CS6 (Adobe, Mountain View, CA, USA).

## Results

**Fig. 2:**
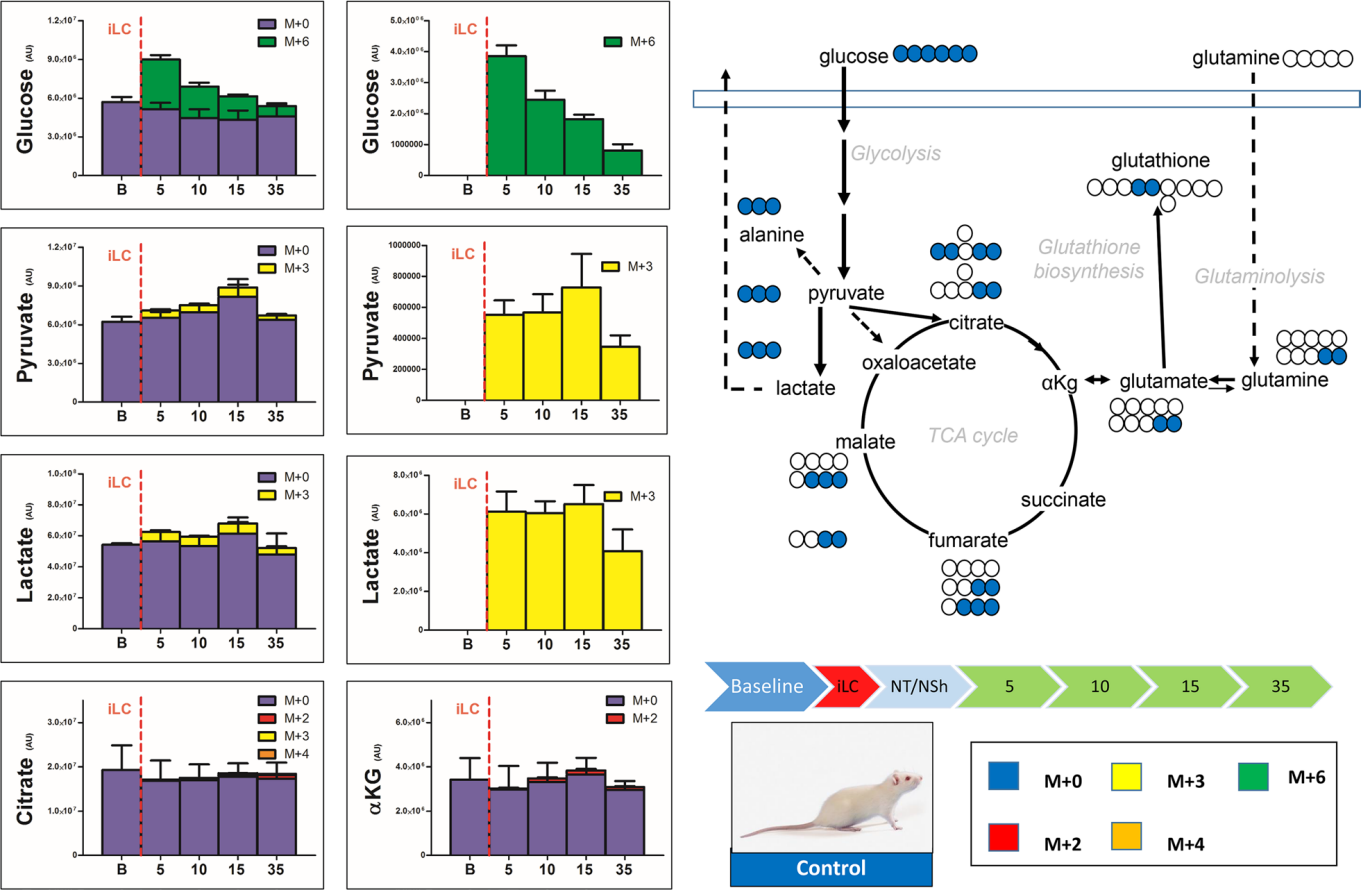
Blood from control rats (no trauma, no hemorrhagic shock) was withdrawn before injection (*baseline* B) of labeled ^13^C-glucose (iLC) and 5, 10, 15 and 35 min after. Metabolites of glycolysis and Krebs cycles were monitored, as they have been previously shown to increase in plasma after trauma/hemorrhagic shock [14]. In *left*, the total levels of the metabolite (integrated peak areas—arbitrary units) are indicated through stacked *bar graphs*, including the unlabeled parent (*blue* M + 0) and heavy isotopologues (either M + 2, M + 3, M + 4 or M + 6 depending on the expected labeling pattern from catabolism of ^13^C-glucose—a schematic overview is provided in the *upper right corner*). In the *right hand panels*, only heavy isotopologues (*red*, *yellow*, *orange*, *green*) are shown. Glucose injection did not affect metabolic profiles, while progressive distribution of heavy carbon atoms was observed for all metabolites. Glycolytic products pyruvate and lactate reached the steady state after 5—15 min from spiked in injection of glucose.

**Fig. 3:**
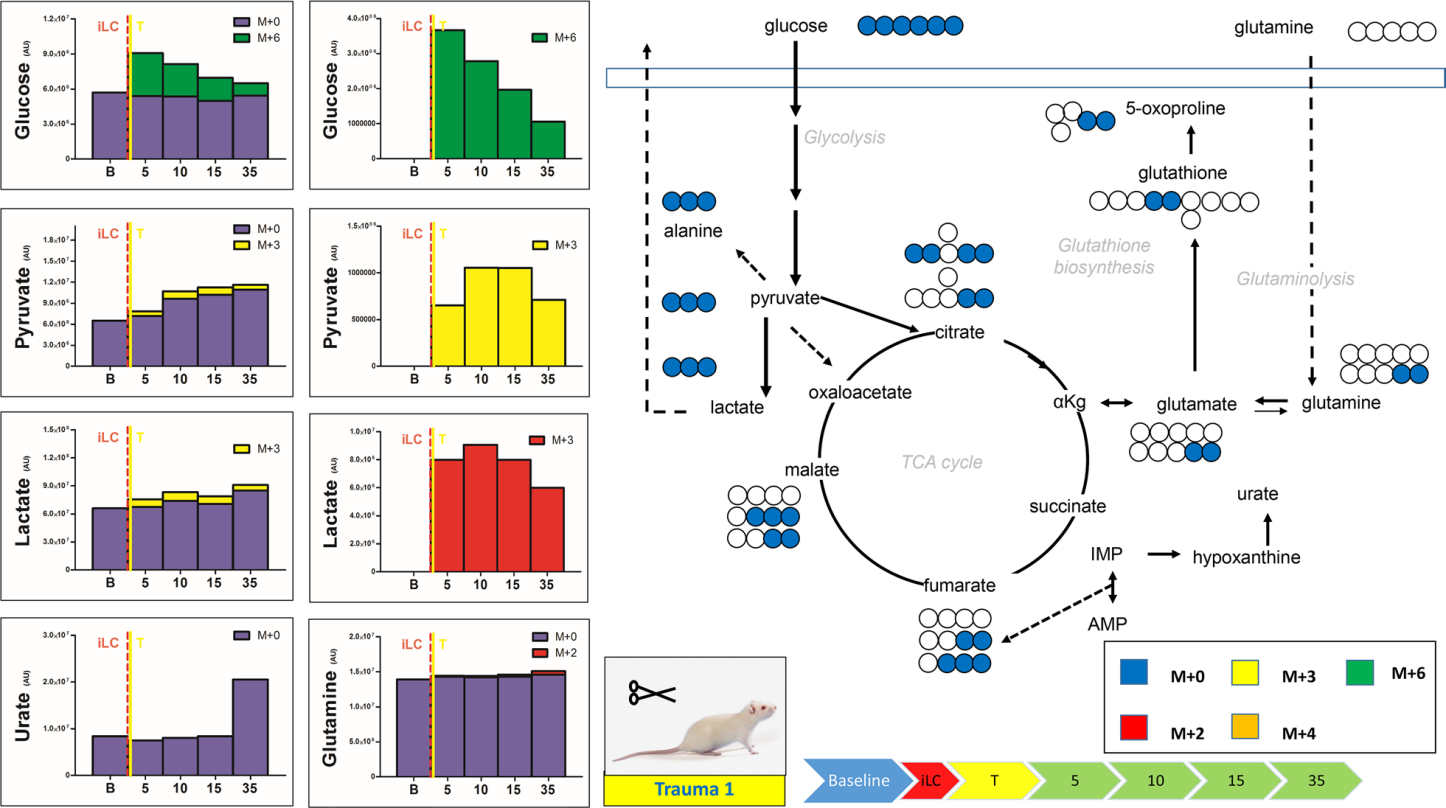
Blood from trauma rats (laparotomy with bowel crush, no hemorrhagic shock) was withdrawn before injection (*baseline* B) of labeled ^13^C-glucose (iLC) preceding trauma. Blood was then collected at 5, 10, 15 and 35 min after iLC. Metabolites of glycolysis and Krebs cycles were monitored, as they have been previously shown to increase in plasma after trauma/hemorrhagic shock [14]. In *left*, the total levels of the metabolite (integrated peak areas—arbitrary units) are indicated through stacked* bar graphs*, including the unlabeled parent (*blue*—M + 0) and heavy isotopologues (either M + 2, M + 3, M + 4 or M + 6 depending on the expected labeling pattern from catabolism of ^13^C-glucose—a schematic overview is provided in the *upper right corner*). In the *right hand panels*, only heavy isotopologues (*red*, *yellow*, *orange*, *green*) are shown. Trauma primed induced urate increases after 35 min, even though metabolic profiles were not affected in general by trauma alone.

**Fig. 4:**
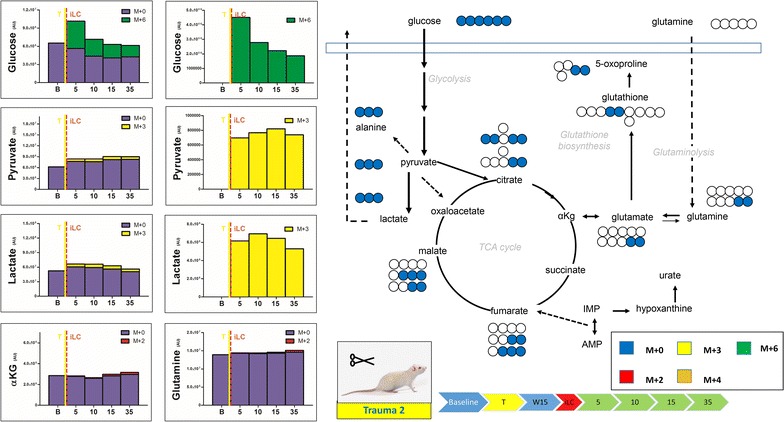
Blood from trauma rats (laparotomy with bowel crush, no hemorrhagic shock) was withdrawn before trauma, preceding injection (*baseline* B) of labeled ^13^C-glucose (iLC). Blood was then collected at 5, 10, 15 and 35 min after iLC. Metabolites of glycolysis and Krebs cycles were monitored, as they have been previously shown to increase in plasma after trauma/hemorrhagic shock [14]. In *left*, the total levels of the metabolite (integrated peak areas—arbitrary units) are indicated through stacked* bar graphs*, including the unlabeled parent (*blue* M + 0) and heavy isotopologues (either M + 2, M + 3, M + 4 or M + 6 depending on the expected labeling pattern from catabolism of ^13^C-glucose—a schematic overview is provided in the *upper right corner*). In the *right hand panels*, only heavy isotopologues (*red*, *yellow*, *orange*, *green*) are shown. Metabolic profiles were not affected by trauma alone.

**Fig. 5:**
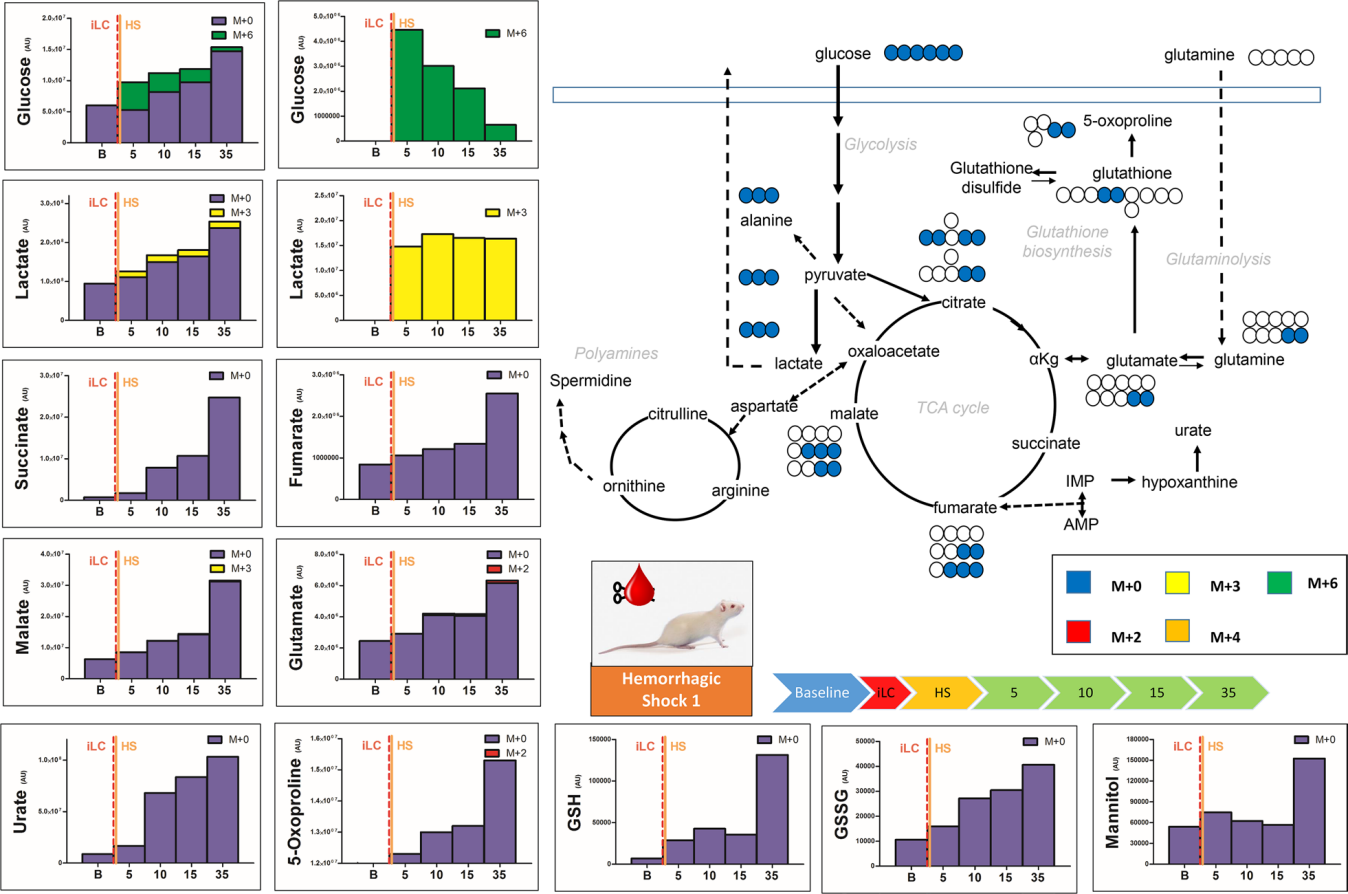
Blood from trauma/hemorrhagic shock rats (laparotomy with bowel crush, with hemorrhagic shock to MAP < 30) was withdrawn before injection (*baseline* B) of labeled ^13^C-glucose (iLC) preceding hemorrhagic shock. Blood was then collected at 5, 10, 15 and 35 min after iLC. Metabolites of glycolysis and Krebs cycles were monitored, as they have been previously shown to increase in plasma after trauma/hemorrhagic shock [14]. In *left*, the total levels of the metabolite (integrated peak areas—arbitrary units) are indicated through stacked *bar graphs*, including the unlabeled parent (*blue* M + 0) and heavy isotopologues (either M + 2, M + 3 or M + 4 depending on the expected labeling pattern from catabolism of ^13^C-glucose—a schematic overview is provided in the *upper right corner*). In the *right hand panels*, only heavy isotopologues (M + 2 = *red*, M + 3 = *yellow*, M + 4 = *orange*, M + 6 = *green*) are shown. As soon as 10 min after iLC, hemorrhagic shock induced accumulation of lactate and unlabeled glucose (indicative of ongoing gluconeogenesis) and late Krebs cycle intermediates (succinate, fumarate, malate), increased levels of glutamate and totally unlabeled urate, polyamines (spermidine), glutathione (either reduced—GSH and oxidized—GSSG), mannitol and citramalate.

**Fig. 6:**
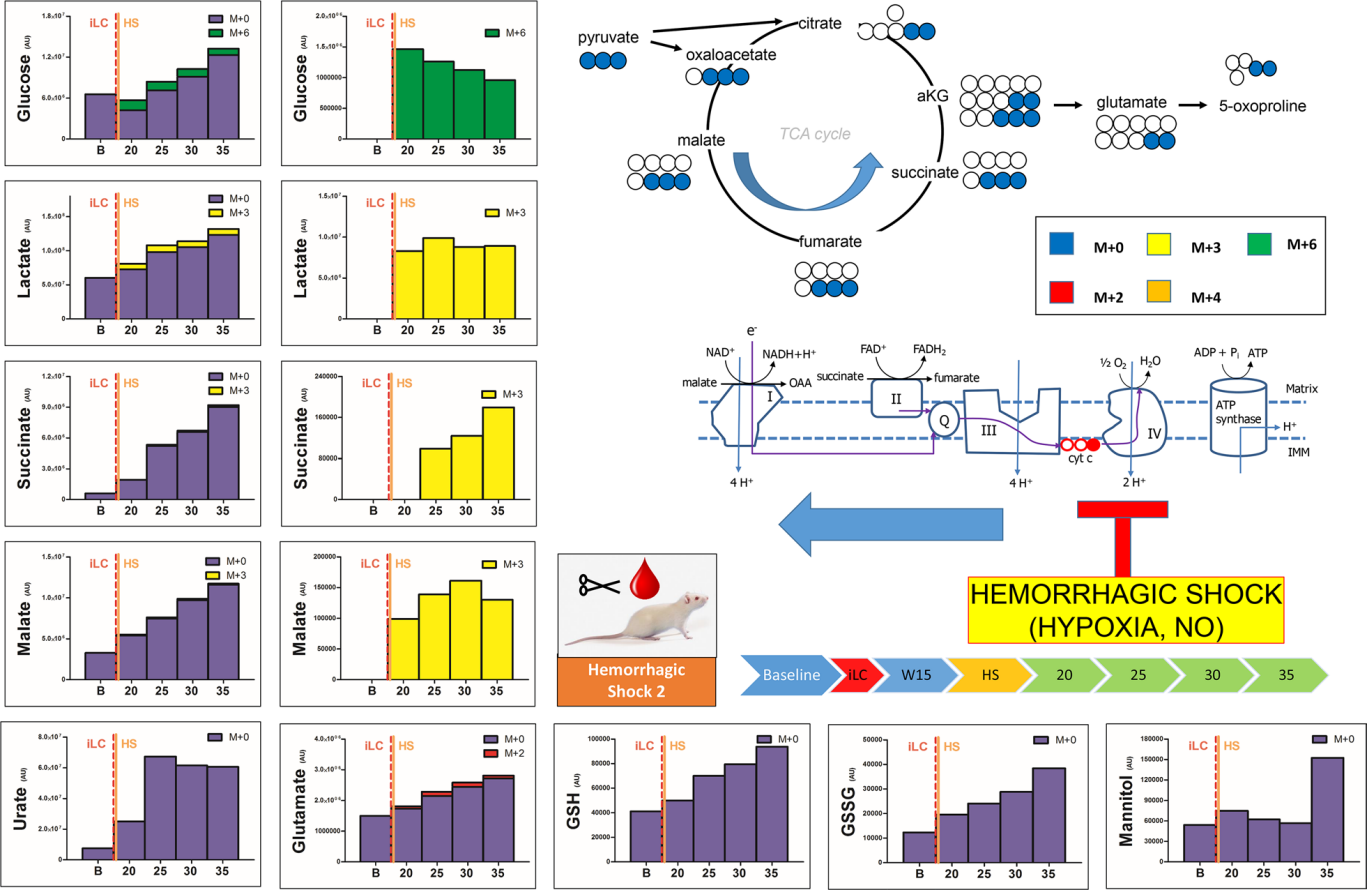
Blood from trauma/hemorrhagic shock rats (laparotomy with bowel crush, with hemorrhagic shock to MAP <30) was withdrawn before injection (*baseline*, B) of labeled ^13^C-glucose (iLC). After waiting for 15 min (W15), hemorrhagic shock was induced to MAP <30 and blood was then collected at 20, 25, 30 and 35 min from iLC. Metabolites of glycolysis and Krebs cycles were monitored, as they have been previously shown to increase in plasma after trauma/hemorrhagic shock [14]. In left, the total levels of the metabolite (integrated peak areas—arbitrary units) are indicated through stacked* bar graphs*, including the unlabeled parent (*blue* M + 0) and heavy isotopologues (either M + 2, M + 3, M + 4, M + 6 depending on the expected labeling pattern from catabolism of ^13^C-glucose). In the *right hand panels*, only heavy isotopologues (*red*, *yellow*, *orange*, *green*) are shown. As soon as 25 min after iLC, hemorrhagic shock induced accumulation of lactate and unlabeled glucose (indicative of ongoing gluconeogenesis) and late Krebs cycle intermediates (succinate, fumarate, malate), increased levels of glutamate and totally unlabeled urate, polyamines (spermidine), glutathione (either reduced—GSH and oxidized—GSSG), mannitol and (minimally labeled) citramalate. M + 3 labeling in malate and succinate is suggestive of malate generation from oxaloacetate obtained via pyruvate carboxylase activity and backwards fluxing of complex I and II to generate malate and succinate in the absence of oxygen as a final electron acceptor (following HS *top right corner*).

**Fig. 7:**
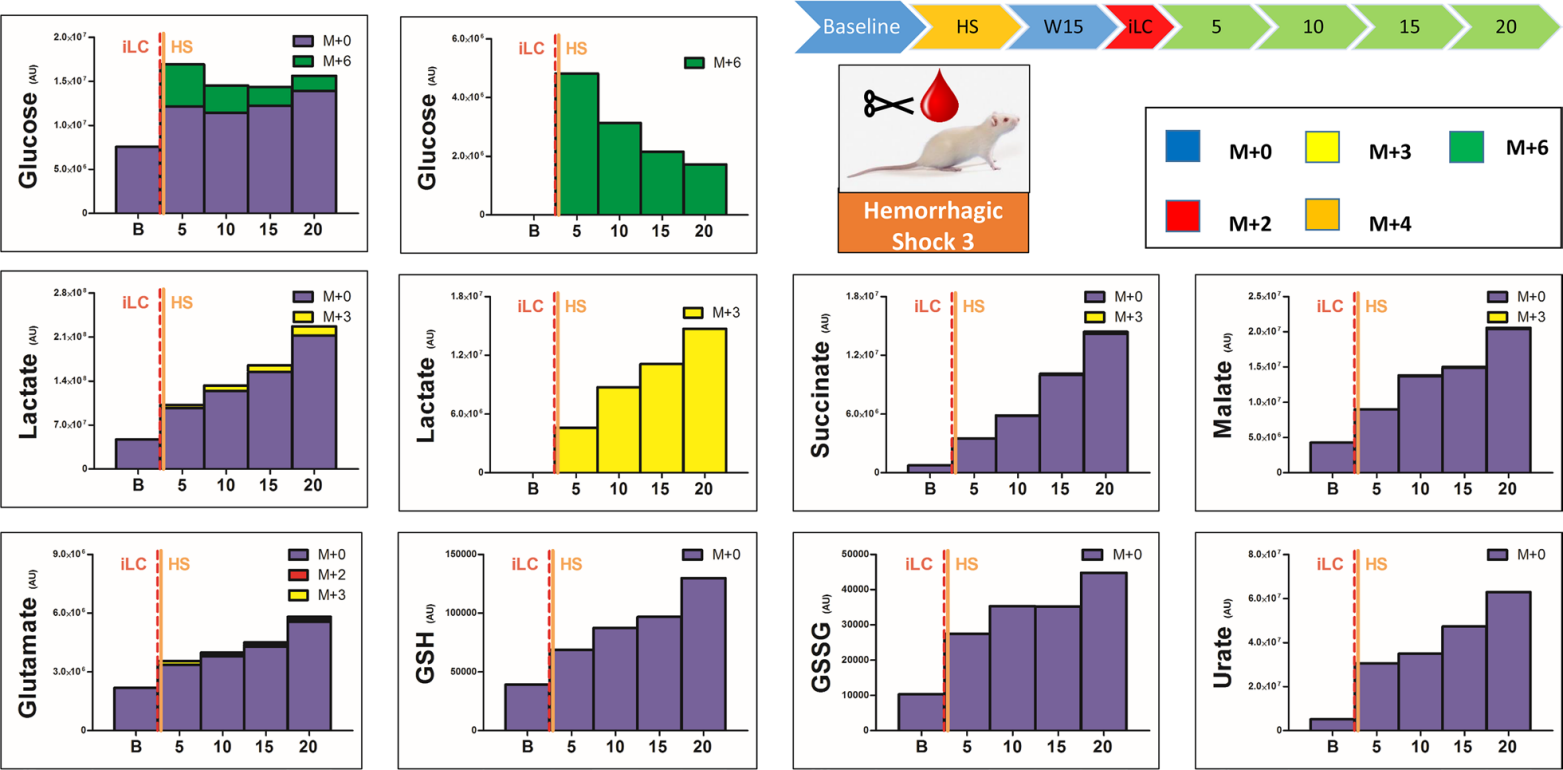
Blood from trauma/hemorrhagic shock rats (laparotomy with bowel crush, with hemorrhagic shock to MAP <30) was withdrawn before hemorrhagic shock (*baseline* B). After waiting for 15 min (W15), injection of labeled ^13^C-glucose was performed and blood was then collected at 5, 10, 15, and 20 min from iLC. Metabolites of glycolysis and Krebs cycles were monitored, as they have been previously shown to increase in plasma after trauma/hemorrhagic shock [14]. In *left*, the total levels of the metabolite (integrated peak areas—arbitrary units) are indicated through stacked *bar graphs*, including the unlabeled parent (*blue* M + 0) and heavy isotopologues (either M + 2, M + 3, M + 4 or M + 6 depending on the expected labeling pattern from catabolism of ^13^C-glucose). In the *right hand panels*, only heavy isotopologues (*red*, *yellow*, *orange*, *green*) are shown. As soon as 5 min after iLC, hemorrhagic shock induced accumulation of lactate and unlabeled glucose (indicative of ongoing gluconeogenesis) and late Krebs cycle intermediates (succinate, fumarate, malate), increased levels of glutamate and totally unlabeled urate, polyamines (spermidine), glutathione (either reduced—GSH and oxidized—GSSG), mannitol and (minimally labeled) citramalate. M + 3 labeling in malate and succinate is suggestive of malate generation from oxaloacetate obtained via pyruvate carboxylase activity and backwards fluxing of complex I and II to generate malate and succinate in the absence of oxygen as a final electron acceptor (following HS *top right corner*).

Plasma metabolomics analyses were performed on eight Sprague–Dawley rats, divided as follows: three control, two trauma and three hemorrhagic shock animals. Injection of labeled ^13^C-glucose (iLC) was performed at different time points, as detailed in Fig. 1. From Figs. 2, 3, 4, 5, 6 and 7 isotopologues were color-coded as indicated in the figure legends (M + 0=blue; M + 2=red; M + 3=yellow; M + 4=orange; M + 6=green). Extended versions of Figs. 2, 3, 4, 5, 6 and 7, including more metabolites and relative heavy isotopologues, are shown in Figs. 1, 2, 3, 4, 5 and 6. iLC alone did not affect plasma metabolic profiles in control rats (Fig. 2). Indeed, kinetic analyses in control rats showed constant total levels of all the tested metabolites for the duration of the experiment without significant change due to biological variability or iLC (Fig. 2, Additional file 1). Heavy carbon tracing in controls showed progressive decrease (metabolism) of heavy labeled glucose (M + 6—Fig. 2), while pyruvate and lactate (M + 3 isotopologues for both trioses) reached steady state after 5 min from the iLC (Fig. 2). Upon correction for natural abundance of ^13^C atoms (1.107%), we found minimum (<5% of total levels) or no labeling of Krebs cycle intermediates (citrate, alpha-ketoglutarate—αKg, succinate, fumarate and malate) in controls (Fig. 2, Additional file 2). Consistently, metabolites involved in transamination reactions (glutamate, alanine) reached the steady state of labeling (equilibrium of catabolism and anabolism) after 10 min from iLC, while the total levels remained constant (Fig. 2). Our control animals confirmed that iLC alone did not create metabolic derangement, and allowed tracing of heavy carbon distributions.

We then tested the metabolic effect of trauma alone on glucose metabolism by injecting a bolus of heavy glucose prior to (Fig. 3) or immediately after (Fig. 4) trauma (no hemorrhagic shock). Injection of heavy carbon prior to trauma did not promote increases in the rates of glycolysis (Fig. 3), with the exception of ~50% increase of pyruvate (both labeled and unlabeled) after 10 min from iLC (Fig. 3). This resulted in only a 10% increase in the levels of lactate at the same time point and 30% lactate increase after 35 min from iLC. The most noticeable change was in the levels of urate (2.4 fold increase from baseline levels—unlabeled, at 35 min from iLC), suggestive of a lag between trauma-primed metabolic changes and activation of purine catabolism. Injection of labeled glucose 15 min after trauma confirmed the results, with pyruvate levels being ~24% higher than the baseline already after 5 min from iLC (Fig. 4). On the other hand, lactate only increased by 10% against baseline levels during the first 20 min, while it went back to baseline levels after 35 min, suggestive of a short-lasting acute response in lactate secondary to trauma alone (Fig. 4). Regardless of timing of iLC, trauma alone was not enough to prime changes at the levels of di- and tricarboxylic acids and amino acids (Additional file 2, Additional file 3).

Conversely, HS induced evident metabolic changes, whether iLC was performed immediately before (Fig. 5, Additional file 4), 15 min before (Fig. 6, Additional file 5) or 15 min into (Fig. 7, Additional file 6) hemorrhage. In particular, iLC during HS resulted in metabolic changes observable at the earliest time point (5 min after iLC—Fig. 7). iLC immediately before HS showed increases in the levels of most metabolites as soon as 10 min after iLC (Fig. 5). By contrast, iLC followed by a 15 min quiescent period before inducing HS resulted in delayed responses (25 min—Fig. 6). Notably, total glucose levels increased up to ~2.5 fold after 35 min from iLC (1.86 ± 0.34 fold versus baseline across all three HS replicates), with ~85% of labeled glucose (M + 6) being consumed (Fig. 5). Lactate levels increased 2.5 fold after 35 min from iLC (58% after 10 min—Fig. 5), a trend that was confirmed when iLC was performed 15 min before HS (twofold after 35 min from iLC—Fig. 6). When iLC was performed during HS, after metabolic responses had already been primed, we observed a twofold increase in lactate compared to baseline values (both labeled and unlabeled) as soon as 5 min from iLC. More significantly, in this model lactate levels rose up to 4.5 fold after 35 min from iLC (Fig. 7), suggesting lasting metabolic derangement following HS, as opposed to the acute transient changes we observed in trauma alone. Pyruvate increases (+41%) were observed only when iLC was spiked during HS (Fig. 7). This suggests fast fluxing of pyruvate to lactate and other metabolic products, such as alanine (+86% increase after 35 min—Fig. 5), a product of pyruvate transamination by glutamate pyruvate transaminase.

In addition, accumulation of Krebs cycle intermediates was significant, and observed early when iLC was given during HS (5 min—Fig. 7). Accumulation was also noted at relatively later time points in pre-shock iLC models (10 min—Fig. 5, or at 25 min—Fig. 6). In particular, late Krebs cycle metabolites succinate, fumarate and malate showed progressive increases after HS (Figs. 5, 6, 7). Succinate for example increased up to 34 fold (median values 19.0 ± 10.2 fold—Fig. 5). In HS rats, malate increases after 35 min from iLC were in the range of 4.76 ± 0.76 fold versus relative baseline values. Provided enough time (15 min) for incubation of labeled glucose, either prior to (Fig. 6) or during HS (Fig. 7), labeling in malate (M + 3) and succinate (M + 3, but not M + 2) was observed. This is suggestive of labeled carbon entering the TCA cycle from carboxylated pyruvate to oxaloacetate, then being converted to malate and succinate via back-fluxing of complex I and II of the electron transport chain due to HS-induced mitochondrial dysfunction (Fig. 6—top right pathway). Still, upon correction for natural abundance, the percentage of labeled succinate was negligible (<5%) in comparison to the total levels of this metabolite (Fig. 7). This is indicative of the majority of this metabolite deriving from other carbon sources than glucose, such as glutamine [15]. Glutaminolysis results in glutamate accumulation, which we observed (+2.5 fold at 35 min versus baseline—Fig. 5). Through transamination reactions, glutamate in converted to aKG, consisted with our non-labeled aKG increases (+60% after 35 min from iLC—Fig. 6). It is also worth noting that while all amino acid levels increased after HS, glutamine levels remained stable across all HS samples, suggestive of catabolism of this metabolite.

HS also promoted the accumulation of glutathione, both GSH and GSSG (Figs. 5, 6, 7), and glutamate/glutathione turn-over product 5-oxoproline (Fig. 5). Labeling of glutamate/glutamine and 5-oxoproline was mostly consistent with labeling in citrate/aKG (M + 2), suggesting acetyl-CoA fluxing through the oxidative branch of the Krebs cycle (Figs. 5, 6, Additional file 4, Additional file 5). However, provided enough time for heavy glucose incubation (iLC 15 min prior to HS) or HS priming of metabolic responses (iLC during HS), M + 3 labeling was observed in both glutamate and 5-oxoproline (Figs. 6, 7), suggestive of reductive fluxing from M + 3 isotopologue of succinate (Fig. 6—top right pathway).

Hemorrhagic shock resulted in earlier and more profound accumulation of urate from purine catabolism (11.6 ± 2.2 fold—Figs. 5, 6, 7) when compared to trauma alone (2.4 fold increase in urate only after 35 min—Fig. 2). Other than purine metabolism, amino acid catabolism deriving from HS-induced proteolysis [14] would result in the accumulation of byproducts of the urea cycle or polyamines [23]. Here we show that unlabeled spermidine accumulation was observed 35 min following HS (Fig. 5).

Finally, unexpected metabolites of potential bacterial origin were recently detected in plasma from our severely injured trauma patients [14], including mannitol and citramalate. In the absence of resuscitation (no mannitol was administered to the rats in any form, including anti-coagulated blood products), we observed HS induced accumulation of unlabeled mannitol (+2.8 ± 0.1 fold—Figs. 5, 6) and citramalate (+2.1 ± 0.3 fold—Figs. 5, 6, 7). Partial labeling (M + 2) of citramalate was observed only when iLC was performed before HS (Additional file 4, Additional file 5).

**Fig. 8:**
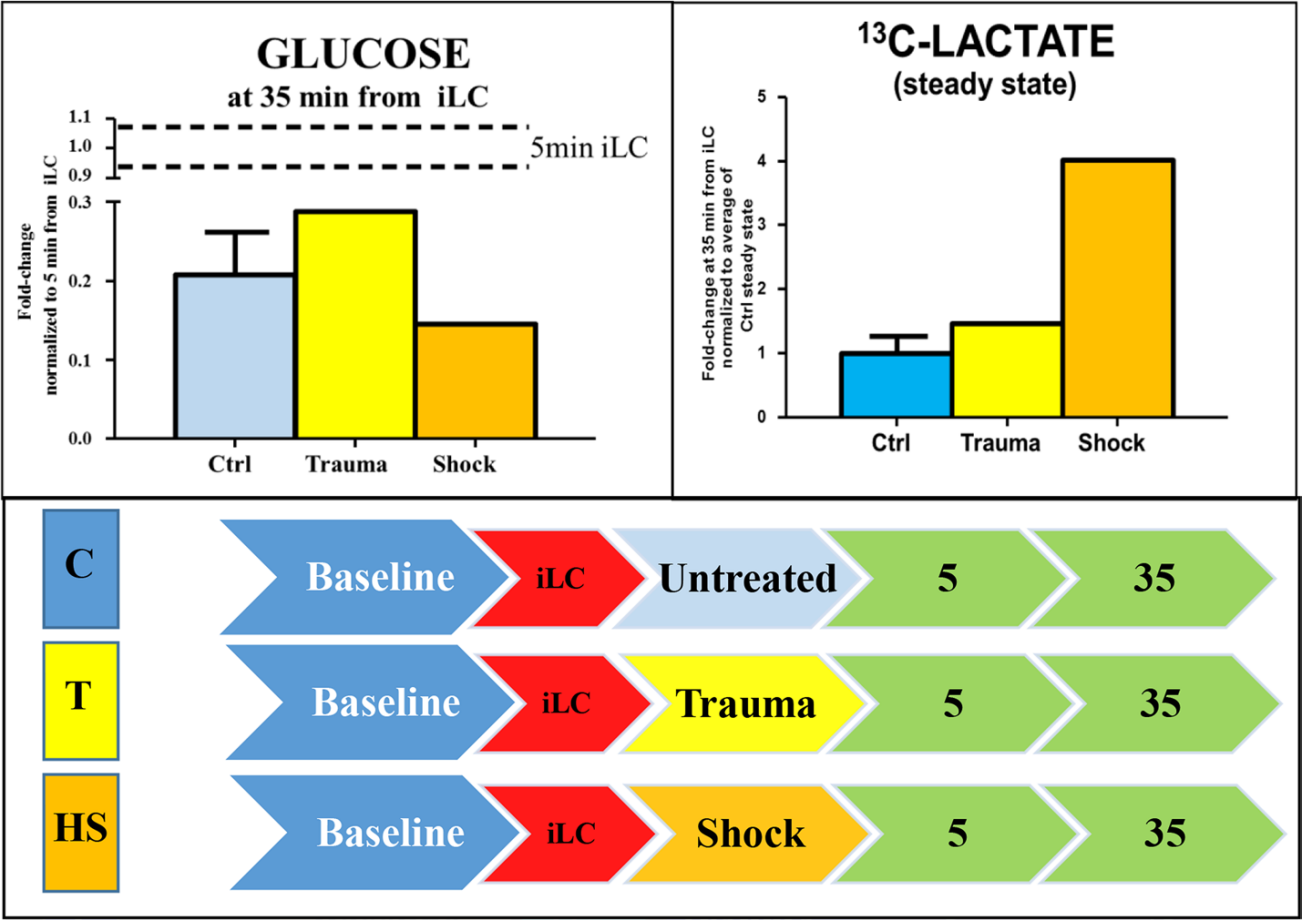
Glucose catabolism (**a**) and steady state levels of labeled (M + 3) lactate (**b**) in plasma from controls (*light blue*), trauma (*yellow*) or hemorrhagic shock (*orange*) rats after 35 min from injection of labeled carbon (iLC). In **A**, heavy labeled glucose (M + 6) detected at 35 min from no treatment (control), trauma or hemorrhage was normalized against the first time point after iLC (considered 100% of the spiked in value). In **b**, values were normalized against the average of steady state levels of labeled lactate detected in controls, and expressed as fold change variations of heavy lactate levels detected in plasma from trauma and hemorrhagic shock rats. Hemorrhagic shock promoted glucose hypercatabolism, while trauma alone reduced fluxes of glucose consumption in comparison to untreated control rats.

While in controls (Fig. 2) and trauma rats (Figs. 3, 4) total glucose levels decreased over time after iLC, in HS group total glucose levels increased (Figs. 5, 6, 7). However, this was not due to decreased catabolism. Indeed, comparative analyses of labeled glucose levels at 35 min after iLC, either in the absence of any treatment (controls), or upon trauma or hemorrhage, resulted in 21 ± 5% of the original levels of spiked in glucose still detectable in control rats, 28% in trauma rats and 14.5% in HS rats, suggesting increased catabolism, other than increased gluconeogenesis/glycogenolysis in the latter group (Fig. 8a). Consistently, four-fold increase in labeled lactate (M + 3) were observed in the HS at the steady state level, in comparison to control counterparts, while only 46% increase were only observed in response to trauma alone (Fig. 8b).

## Discussion

Application of MS-metabolomic technologies to the field of trauma surgery holds great potential in that it promises to expand our understanding of the metabolic staging after critical injury [12, 24]. Further, it can help us identify the mechanistic links between metabolic changes and observed post-injury clinical sequelae, such as inflammation and coagulopathy. It also provides hypothesis generating data indicating unexpected plasma metabolic signatures of T/HS, as in the case of metabolites of potential bacterial origin [14]. Finally, metabolomics can help elucidating the differential metabolic consequences of trauma versus hemorrhagic shock.

It is recognized that acid–base disturbances are common in critically ill or severely injured patients [25]. Metabolic acidosis associated with trauma and HS is thought to be largely a result of lactate production from anaerobic metabolism associated with hypotension and hypoperfusion. Traditional assessment to identify the presence and severity of acidosis includes calculation of lactate levels, base deficit and the anion gap. Investigations have shown that this conventional assessment of acidosis at admission is a marker of injury severity and predicts mortality [25]. However, others suggest inconsistency in the predictive value of these conventional assessments in that they may be confounded by hypoalbuminemia, elevated PaCO_2_, and unmeasured anions [5, 6]. Additionally the severity of acidosis within minutes of injury may not be fully explained by anaerobic metabolism and lactate production alone. It has been demonstrated that unmeasured ions are common contributors to metabolic acidosis in trauma patients admitted to the intensive care unit [6]. These ions can be assessed by a physiochemical approach (SIG) to determine their contribution to acid–base milieu. However the SIG mathematical equation fails to identify the specific contributing anions thus at best it is another estimation without providing specific resuscitation targets.

Recent technologic advances in MS instruments have afforded the sensitivity and specificity to expand the metabolomic coverage when compared to pioneering NMR studies [8, 9, 26]. Also, MS-metabolomics allows for tracing experiments [16] that determine how metabolic substrates are differentially utilized after trauma and hemorrhage, in like fashion to what was recently proposed for ischemic/reperfusion injury [15]. Such a workflow is relevant in that it affords specific, high-throughput analysis of comprehensive metabolomes, opening a window on dynamic fluxes through metabolic pathways. We can now design large-scale clinical trials to determine patient and trauma-specific metabolic responses to critical injury in an attempt to outline tailored resuscitative strategies.

In this preliminary study, we adopted a U-^13^C-glucose labeling strategy in a rat model of T/HS [20] to define glucose metabolism and substrate flux through glycolysis and the Krebs cycle following trauma or hemorrhagic shock. Our results, complementing steady state observations in humans and rats after severe HS [14, 18], demonstrate that trauma alone primes a transient hyper-catabolic state (lactate increase, purine catabolism), without altering amino acid metabolism or plasma homeostasis of di- and tri-carboxylates. Conversely, HS instigates metabolic changes not observed in trauma alone. Above all, HS promotes glucose consumption despite overall increase in the total levels of glucose. Indeed, HS plasma, but not trauma or control plasma, is characterized by higher levels of glucose, which is consistent with an insulin-resistant or traumatic diabetes-like metabotype [12]. However, injection of heavy glucose immediately prior to or 15 min before induction of HS corresponds to lactate levels reaching the steady state at the earliest time points assessed. On the other hand, injection of ^13^C-glucose 15 min after HS corresponds to a progressive accumulation of labeled lactate, suggesting that a glycolytic phenotype ensues only later upon HS after an early hypocatabolic phase (ebb-like phenotype). This is consistent with previous studies describing metabolic staging after trauma in humans and rats.

One of the more profound examples of HS-specific metabolic derangement is the post-shock accumulation of non-lactate acidotic metabolites, including succinate, fumarate and malate. Despite the preliminary nature of the results, here we show that these metabolites are mostly unlabeled (not derived from glucose) and when labeled, show isotopologue distributions consistent with back fluxing from pyruvate-derived oxaloacetate. These results are consistent with electron transport chain uncoupling and back-fluxing to malate and succinate (instead of fluxing to oxaloacetate and fumarate) at the complex I and II level. This is in agreement with recent evidence from animal models of ischemia/reperfusion injury [15], and expand upon the previously hypothesized role of pyruvate carboxylate deficiency in congenital lactate acidosis [27]. On the other hand, >95% of the levels of these metabolites were unlabeled, suggesting that they might be generated from other carbon sources, such as fatty acids or amino acid metabolism [14]. In parallel to most recent advancements in cancer research [16] and consistent with ischemia/reperfusion injury models [15], glutaminolysis represents the most likely explanatory mechanism. Impaired glutaminolysis would explain both the increase in unlabeled succinate (bottleneck at the complex II level) and the increase in the levels of glutamate and the glutathione pool observed in response to HS, but not trauma alone. Alternatively, fatty acid or amino acid catabolism might be alternative explanations that will be tested in future experiments (e.g. bolus injections of ^13^C-palmitate instead of heavy glucose in the present model).

While the presence of Krebs cycle intermediates in plasma from T/HS rats [18] and human trauma patients [14] is anticipated, appearance of the M + 3 labeling pattern in these compounds is suggestive either of mitolysis or cell lysis and release of these compounds in plasma as a consequence to HS. Notably, only some of the enzymes involved in these reactions (malate dehydrogenase) have been reported in plasma of T/HS patients as a result of cell lysis [28]. Of note, when in plasma, these proteins may not exert their normal enzymatic activity when circulating in an extracellular environment [28]. Consistently, plasma increases in the levels of some carboxylic acids, like succinate (consistent with steady state observations in humans and rats after severe HS [14, 18]), might play unexpected biological functions such as acting as paracrine immunomodulatory signaling promoting macrophage activation [29], and thus potentially mediate pro-inflammatory mechanisms driving distal organ (e.g. lung) injury after trauma and hemorrhage.

Urate was the metabolite showing the highest fold change increases after HS, an observation consistent with our previous metabolomic studies on alternative rat models of trauma and deep shock [18]. This is significant as debate surrounds urate as an anti-oxidant or pro-oxidant compound, while its conversion to allantoin might be promoted by scavenging of reactive oxygen species [18], or triggered by the enzymatic activity of uricase, an enzyme that is not functional in humans and great apes. Further, purine catabolism is theorized to contribute to vasodilation, as circulating adenosine levels have recently been tied to the activation of Adora2b (adenosine receptor 2b)-dependent cascades, promoting the inhibition pro-inflammatory cascades triggering organ injury (e.g. kidney) [30] and adaptation to hypoxemia [31].

We recently reported the detection of increased levels of potentially non-mammalian metabolites in plasma from severely injured trauma patients [14]. In that study, metabolites such as mannitol and citramalate were attributed to the transfusion of blood products (packed red cells) or hemolysis because of previously documented detection of citramalate in human red blood cells [32]. Despite these explanations, preliminary metabolomics results were intriguing in that they provided an alternative rationale linking metabolites derived from the gut microbiome to post-T/HS sequelae without the need for actual bacterial translocation (consistent with controversial data on showing no direct marker of bacterial translocation in trauma [33]). To confirm and expand upon this hypothesis, here we performed preliminary tracing experiments to test whether labeling incorporation from heavy glucose could be observed in the levels of these metabolites. Our results demonstrate that only HS, not trauma alone, promoted the accumulation of mannitol and citramalate, and that only the latter showed very minor (≪5%) incorporation of labeling from glucose, provided enough time was warranted for the incorporation heavy labeled carbons from glucose into this metabolite. This is suggestive that plasma elevation in the levels of these metabolites is a slow occurring event in comparison to the fast metabolic adaptations involving glycolytic and tri-carboxylic acid homeostasis. Further studies will investigate the origin of these compounds, for example through the adoption of T/HS models using aseptic rats.

## Conclusion

In this study we used intravenously injected ^13^C-glucose labeling to document the kinetics of glycolytic metabolism following trauma and HS. Injection of the labeled substrate was performed prior to or after trauma or HS in order to monitor isotopologue distribution patterns in glycolytic products and di- and tri-carboxylic acids. As a result we confirmed that while trauma promotes transient metabolic change, HS provokes sustained metabolic aberrations. Further, significant changes were seen within 5–10 min of iLC.

Our results, though preliminary, suggest that HS instigates increased rates of glycolysis and the accumulation of pyruvate and lactate, a phenomenon only primed by trauma. Also, HS primed minor labeling accumulation of Krebs cycle intermediates, especially malate, fumarate and succinate. However, isotopologue distributions (M + 3 labeling) suggested back-fluxing through complex I and II of the electron transport chain in the hemorrhagic shock state, or fueling of the Krebs cycle by non-glucose carbon sources. Absent or minimal labeling of glutamate, glutathione (either reduced—GSH or disulfide—GSSG) was also observed despite significant increases following HS. In the light of previous observations in ischemia/reperfusion injury [15], this evidence suggests that upon HS fluxing occurs from non-glucose carbon sources, such as glutamine. Accumulating urate, a byproduct of purine catabolism and a potential contributor to oxidative stress, was primed by trauma, but exacerbated by HS.

The present preliminary study provides the foundation for additional studies employing heavy labeled substrates to monitor amino acid (glutamine), fatty acid and purine (adenosine) metabolism under trauma and hemorrhagic shock conditions. Results from additional labeling studies on a larger biological population will confirm, complement and expand data from the present study and inform the interpretation of plasma metabolomics analyses of hundreds of human subjects enrolled in our ongoing clinical trials (COMBAT and TACTIC) [34, 35]. Ultimately, these mechanistic details have the potential to drive the design of tailored resuscitative strategies based on real-time, high-throughput metabolomic analyses of individual trauma patients, an unprecedented opportunity made amenable by recent innovation in mass spectrometry-based analyses. The final goal will be to design resuscitative interventions directed at restoring metabolic homeostasis, as to prevent highly morbid and even deadly post-shock events instigated by metabolic deregulations.

## Additional files

**Additional file 1**. Blood from control rats (no trauma, no hemorrhagic shock) was withdrawn before injection (*baseline* B) of labeled ^13^C-glucose (iLC) and 5, 10, 15 and 35 min after. Metabolites of glycolysis and Krebs cycles were monitored, as they have been previously shown to increase in plasma after trauma/hemorrhagic shock [14]. In *left*, the total levels of the metabolite (integrated peak areas—arbitrary units) are indicated through stacked *bar graphs*, including the unlabeled parent (*blue* M + 0) and heavy isotopologues (either M + 2, M + 3, M + 4 or M + 6 depending on the expected labeling pattern from catabolism of ^13^C-glucose—a schematic overview is provided in the *upper right corner*). In the *right hand panels*, only heavy isotopologues (*red*, *yellow*, *orange*, *green*) are shown. Glucose injection did not affect metabolic profiles, while progressive distribution of heavy carbon atoms was observed for all metabolites. Glycolytic products pyruvate and lactate reached the steady state after 5—15 min from spiked in injection of glucose. This figure is an extended version of the in manuscript Fig. 2.
**Additional file 2**. Blood from trauma rats (laparotomy with bowel crush, no hemorrhagic shock) was withdrawn before injection (*baseline* B) of labeled ^13^C-glucose (iLC) preceding trauma. Blood was then collected at 5, 10, 15 and 35 min after iLC. Metabolites of glycolysis and Krebs cycles were monitored, as they have been previously shown to increase in plasma after trauma/hemorrhagic shock [14]. In *left*, the total levels of the metabolite (integrated peak areas—arbitrary units) are indicated through stacked* bar graphs*, including the unlabeled parent (*blue*—M + 0) and heavy isotopologues (either M + 2, M + 3, M + 4 or M + 6 depending on the expected labeling pattern from catabolism of ^13^C-glucose—a schematic overview is provided in the *upper right corner*). In the *right hand panels*, only heavy isotopologues (*red*, *yellow*, *orange*, *green*) are shown. Trauma primed induced urate increases after 35 min, even though metabolic profiles were not affected in general by trauma alone. This figure is an extended version of the in manuscript Fig. 3.
**Additional file 3**. Blood from trauma rats (laparotomy with bowel crush, no hemorrhagic shock) was withdrawn before trauma, preceding injection (*baseline* B) of labeled ^13^C-glucose (iLC). Blood was then collected at 5, 10, 15 and 35 min after iLC. Metabolites of glycolysis and Krebs cycles were monitored, as they have been previously shown to increase in plasma after trauma/hemorrhagic shock [14]. In *left*, the total levels of the metabolite (integrated peak areas—arbitrary units) are indicated through stacked* bar graphs*, including the unlabeled parent (*blue* M + 0) and heavy isotopologues (either M + 2, M + 3, M + 4 or M + 6 depending on the expected labeling pattern from catabolism of ^13^C-glucose—a schematic overview is provided in the *upper right corner*). In the *right hand panels*, only heavy isotopologues (*red*, *yellow*, *orange*, *green*) are shown. Metabolic profiles were not affected by trauma alone. This figure is an extended version of the in manuscript Fig. 4.
**Additional file 4**. Blood from trauma/hemorrhagic shock rats (laparotomy with bowel crush, with hemorrhagic shock to MAP < 30) was withdrawn before injection (*baseline* B) of labeled ^13^C-glucose (iLC) preceding hemorrhagic shock. Blood was then collected at 5, 10, 15 and 35 min after iLC. Metabolites of glycolysis and Krebs cycles were monitored, as they have been previously shown to increase in plasma after trauma/hemorrhagic shock [14]. In *left*, the total levels of the metabolite (integrated peak areas—arbitrary units) are indicated through stacked *bar graphs*, including the unlabeled parent (*blue* M + 0) and heavy isotopologues (either M + 2, M + 3 or M + 4 depending on the expected labeling pattern from catabolism of ^13^C-glucose—a schematic overview is provided in the *upper right corner*). In the *right hand panels*, only heavy isotopologues (M + 2 = *red*, M + 3 = *yellow*, M + 4 = *orange*, M + 6 = *green*) are shown. As soon as 10 min after iLC, hemorrhagic shock induced accumulation of lactate and unlabeled glucose (indicative of ongoing gluconeogenesis) and late Krebs cycle intermediates (succinate, fumarate, malate), increased levels of glutamate and totally unlabeled urate, polyamines (spermidine), glutathione (either reduced—GSH and oxidized—GSSG), mannitol and citramalate. This figure is an extended version of the in manuscript Fig. 5.
**Additional file 5**. Blood from trauma/hemorrhagic shock rats (laparotomy with bowel crush, with hemorrhagic shock to MAP <30) was withdrawn before injection (*baseline*, B) of labeled ^13^C-glucose (iLC). After waiting for 15 min (W15), hemorrhagic shock was induced to MAP <30 and blood was then collected at 20, 25, 30 and 35 min from iLC. Metabolites of glycolysis and Krebs cycles were monitored, as they have been previously shown to increase in plasma after trauma/hemorrhagic shock [14]. In left, the total levels of the metabolite (integrated peak areas—arbitrary units) are indicated through stacked* bar graphs*, including the unlabeled parent (*blue* M + 0) and heavy isotopologues (either M + 2, M + 3, M + 4, M + 6 depending on the expected labeling pattern from catabolism of ^13^C-glucose). In the *right hand panels*, only heavy isotopologues (*red*, *yellow*, *orange*, *green*) are shown. As soon as 25 min after iLC, hemorrhagic shock induced accumulation of lactate and unlabeled glucose (indicative of ongoing gluconeogenesis) and late Krebs cycle intermediates (succinate, fumarate, malate), increased levels of glutamate and totally unlabeled urate, polyamines (spermidine), glutathione (either reduced—GSH and oxidized—GSSG), mannitol and (minimally labeled) citramalate. M + 3 labeling in malate and succinate is suggestive of malate generation from oxaloacetate obtained via pyruvate carboxylase activity and backwards fluxing of complex I and II to generate malate and succinate in the absence of oxygen as a final electron acceptor (following HS *top right corner*). This figure is an extended version of the in manuscript Fig. 6.
**Additional file 6**. Blood from trauma/hemorrhagic shock rats (laparotomy with bowel crush, with hemorrhagic shock to MAP <30) was withdrawn before hemorrhagic shock (*baseline* B). After waiting for 15 min (W15), injection of labeled ^13^C-glucose was performed and blood was then collected at 5, 10, 15, and 20 min from iLC. Metabolites of glycolysis and Krebs cycles were monitored, as they have been previously shown to increase in plasma after trauma/hemorrhagic shock [14]. In *left*, the total levels of the metabolite (integrated peak areas—arbitrary units) are indicated through stacked *bar graphs*, including the unlabeled parent (*blue* M + 0) and heavy isotopologues (either M + 2, M + 3, M + 4 or M + 6 depending on the expected labeling pattern from catabolism of ^13^C-glucose). In the *right hand panels*, only heavy isotopologues (*red*, *yellow*, *orange*, *green*) are shown. As soon as 5 min after iLC, hemorrhagic shock induced accumulation of lactate and unlabeled glucose (indicative of ongoing gluconeogenesis) and late Krebs cycle intermediates (succinate, fumarate, malate), increased levels of glutamate and totally unlabeled urate, polyamines (spermidine), glutathione (either reduced—GSH and oxidized—GSSG), mannitol and (minimally labeled) citramalate. M + 3 labeling in malate and succinate is suggestive of malate generation from oxaloacetate obtained via pyruvate carboxylase activity and backwards fluxing of complex I and II to generate malate and succinate in the absence of oxygen as a final electron acceptor (following HS *top right corner*). This figure is an extended version of the in manuscript Fig. 7.
