# Supplementary material for: Trauma/hemorrhagic shock instigates aberrant metabolic flux through glycolytic pathways, as revealed by preliminary 13C-glucose labeling metabolomics
Source: J Transl Med. 2015 Aug 5;13:253. doi: 10.1186/s12967-015-0612-z (PMC4523956; doi:10.1186/s12967-015-0612-z)
Supplement: Supplementary file 1 — Additional file 1. Blood from control rats (no trauma, no hemorrhagic shock) was withdrawn before injection (baseline B) of labeled 13C-glucose (iLC) and 5, 10, 15 and 35 min after. Metabolites of glycolysis and Krebs cycles were monitored, as they have been previously shown to increase in plasma after trauma/hemorrhagic shock [14]. In left, the total levels of the metabolite (integrated peak areas—arbitrary units) are indicated through stacked bar graphs, including the unlabeled parent (blue M + 0) and heavy isotopologues (either M + 2, M + 3, M + 4 or M + 6 depending on the expected labeling pattern from catabolism of 13C-glucose—a schematic overview is provided in the upper right corner). In the right hand panels, only heavy isotopologues (red, yellow, orange, green) are shown. Glucose injection did not affect metabolic profiles, while progressive distribution of heavy carbon atoms was observed for all metabolites. Glycolytic products pyruvate and lactate reached the steady state after 5—15 min from spiked in injection of glucose. This figure is an extended version of the in manuscript Fig. 2. [file 12967_2015_612_MOESM1_ESM.pdf]

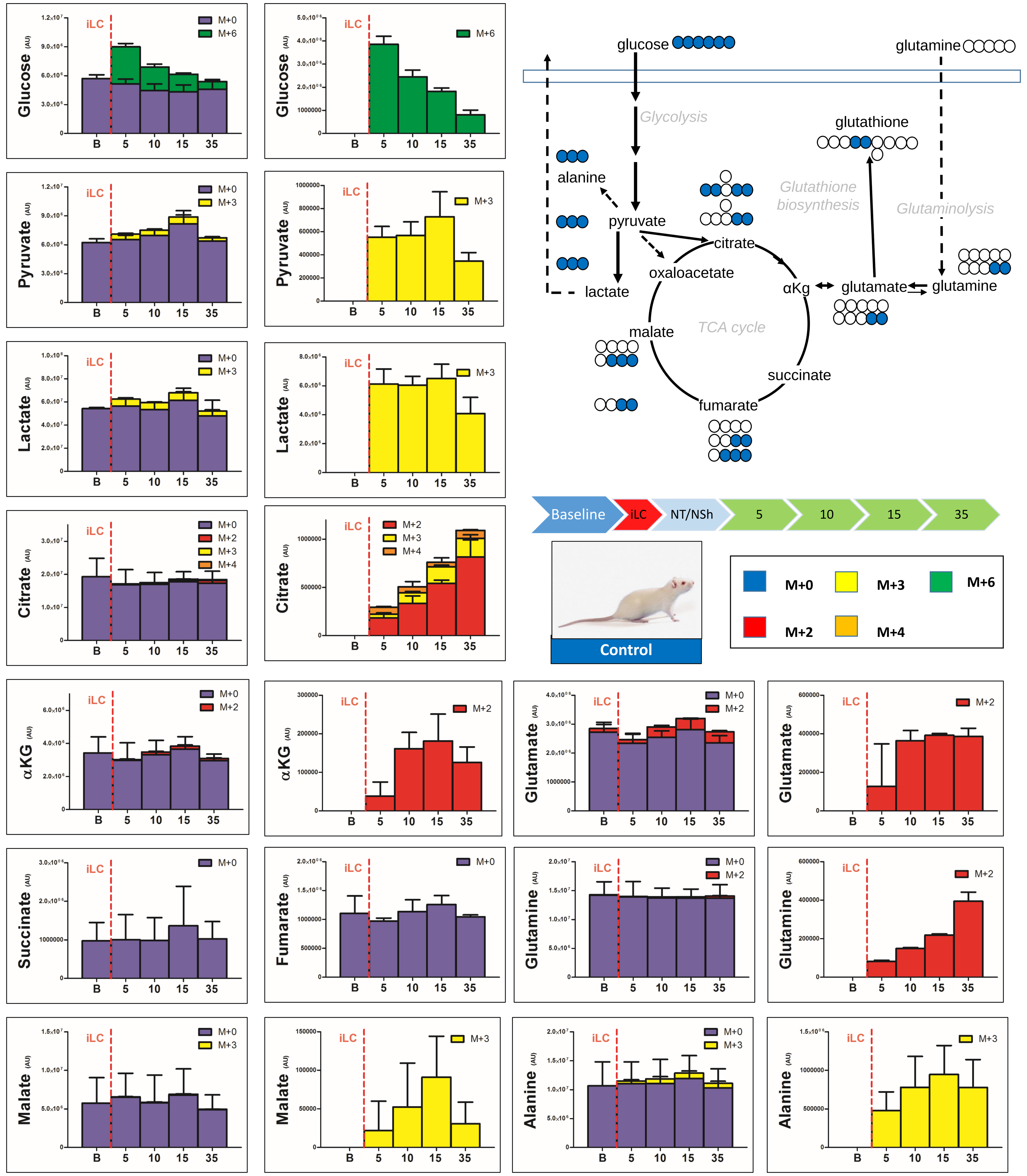

Additional Figure 1 - Blood from control rats (no trauma, no hemorrhagic shock) was withdrawn before injection (baseline – B) of labeled ^{13}C-glucose (iLC) and 5, 10, 15 and 35 minutes after. Metabolites of glycolysis and Krebs cycles were monitored, as they have been previously shown to increase in plasma after trauma/hemorrhagic shock [14]. In left, the total levels of the metabolite (integrated peak areas – arbitrary units) are indicated through stacked bar graphs, including the unlabeled parent (blue – M+0) and heavy isotopologues (either M+2, M+3, M+4 or M+6 depending on the expected labeling pattern from catabolism of ^{13}C-glucose – a schematic overview is provided in the upper right corner). In the right hand panels, only heavy isotopologues (red, yellow, orange, green) are shown. Glucose injection did not affect metabolic profiles, while progressive distribution of heavy carbon atoms was observed for all metabolites. Glycolytic products pyruvate and lactate reached the steady state after 5-15 minutes from spiked in injection of glucose. Extended version of in text Figure 2.
