# Supplementary material for: Trauma/hemorrhagic shock instigates aberrant metabolic flux through glycolytic pathways, as revealed by preliminary 13C-glucose labeling metabolomics
Source: J Transl Med. 2015 Aug 5;13:253. doi: 10.1186/s12967-015-0612-z (PMC4523956; doi:10.1186/s12967-015-0612-z)
Supplement: Supplementary file 3 — Additional file 3. Blood from trauma rats (laparotomy with bowel crush, no hemorrhagic shock) was withdrawn before trauma, preceding injection (baseline B) of labeled 13C-glucose (iLC). Blood was then collected at 5, 10, 15 and 35 min after iLC. Metabolites of glycolysis and Krebs cycles were monitored, as they have been previously shown to increase in plasma after trauma/hemorrhagic shock [14]. In left, the total levels of the metabolite (integrated peak areas—arbitrary units) are indicated through stacked bar graphs, including the unlabeled parent (blue M + 0) and heavy isotopologues (either M + 2, M + 3, M + 4 or M + 6 depending on the expected labeling pattern from catabolism of 13C-glucose—a schematic overview is provided in the upper right corner). In the right hand panels, only heavy isotopologues (red, yellow, orange, green) are shown. Metabolic profiles were not affected by trauma alone. This figure is an extended version of the in manuscript Fig. 4. [file 12967_2015_612_MOESM3_ESM.pdf]

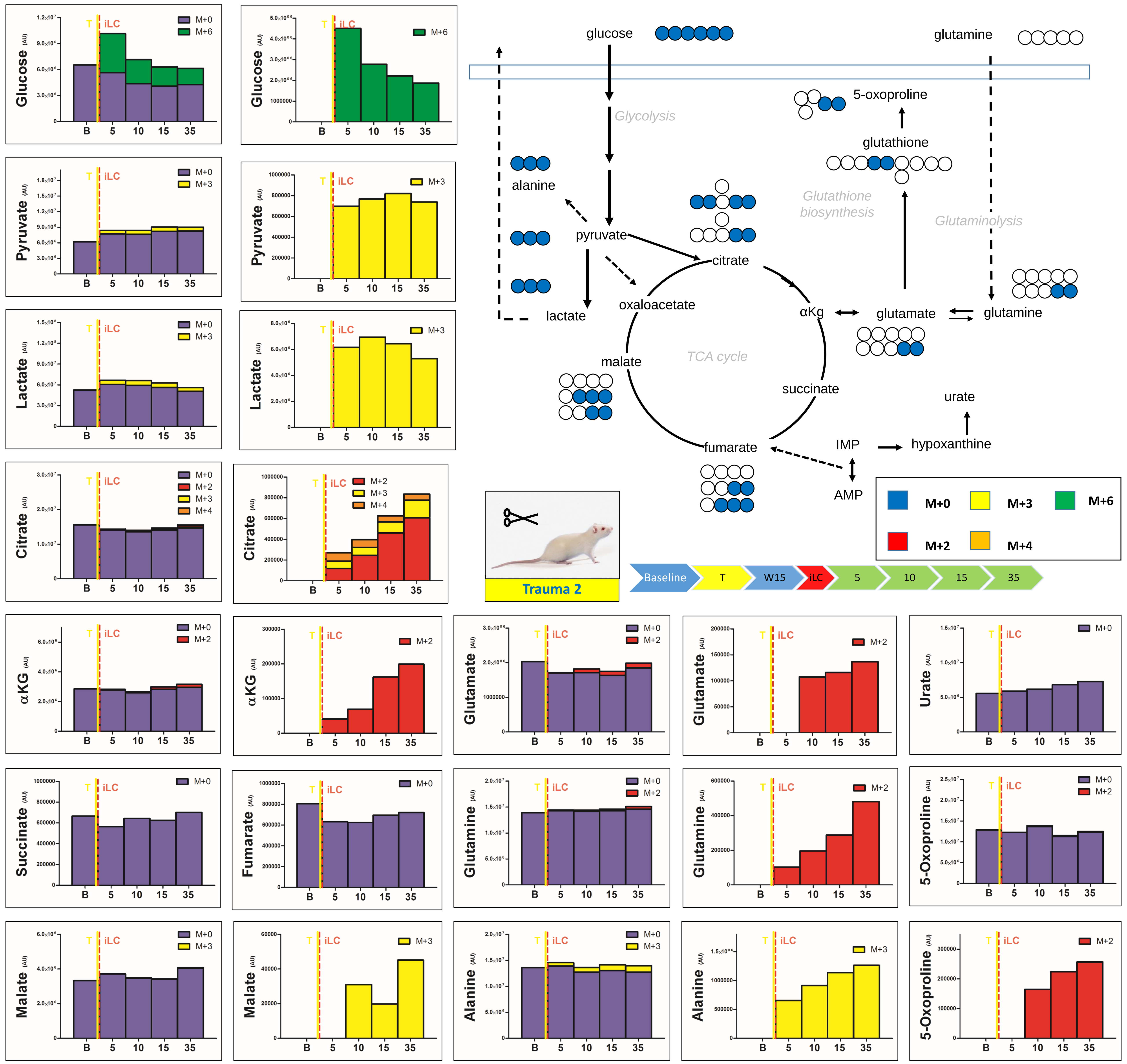

Additional Figure 3 - Blood from trauma rats (laparotomy with bowel crush, no hemorrhagic shock) was withdrawn before trauma, preceding injection (baseline – B) of labeled 13C-glucose (iLC). Blood was then collected at 5, 10, 15 and 35 minutes after iLC. Metabolites of glycolysis and Krebs cycles were monitored, as they have been previously shown to increase in plasma after trauma/hemorrhagic shock [14]. In left, the total levels of the metabolite (integrated peak areas – arbitrary units) are indicated through stacked bar graphs, including the unlabeled parent (blue – M+0) and heavy isotopologues (either M+2, M+3, M+4 or M+6 depending on the expected labeling pattern from catabolism of 13C-glucose – a schematic overview is provided in the upper right corner). In the right hand panels, only heavy isotopologues (red, yellow, orange, green) are shown. Metabolic profiles were not affected by trauma alone. Extended version of in text Figure 4.
