# Supplementary material for: Trauma/hemorrhagic shock instigates aberrant metabolic flux through glycolytic pathways, as revealed by preliminary 13C-glucose labeling metabolomics
Source: J Transl Med. 2015 Aug 5;13:253. doi: 10.1186/s12967-015-0612-z (PMC4523956; doi:10.1186/s12967-015-0612-z)
Supplement: Supplementary file 5 — Additional file 5. Blood from trauma/hemorrhagic shock rats (laparotomy with bowel crush, with hemorrhagic shock to MAP <30) was withdrawn before injection (baseline, B) of labeled 13C-glucose (iLC). After waiting for 15 min (W15), hemorrhagic shock was induced to MAP <30 and blood was then collected at 20, 25, 30 and 35 min from iLC. Metabolites of glycolysis and Krebs cycles were monitored, as they have been previously shown to increase in plasma after trauma/hemorrhagic shock [14]. In left, the total levels of the metabolite (integrated peak areas—arbitrary units) are indicated through stacked bar graphs, including the unlabeled parent (blue M + 0) and heavy isotopologues (either M + 2, M + 3, M + 4, M + 6 depending on the expected labeling pattern from catabolism of 13C-glucose). In the right hand panels, only heavy isotopologues (red, yellow, orange, green) are shown. As soon as 25 min after iLC, hemorrhagic shock induced accumulation of lactate and unlabeled glucose (indicative of ongoing gluconeogenesis) and late Krebs cycle intermediates (succinate, fumarate, malate), increased levels of glutamate and totally unlabeled urate, polyamines (spermidine), glutathione (either reduced—GSH and oxidized—GSSG), mannitol and (minimally labeled) citramalate. M + 3 labeling in malate and succinate is suggestive of malate generation from oxaloacetate obtained via pyruvate carboxylase activity and backwards fluxing of complex I and II to generate malate and succinate in the absence of oxygen as a final electron acceptor (following HS top right corner). This figure is an extended version of the in manuscript Fig. 6. [file 12967_2015_612_MOESM5_ESM.pdf]

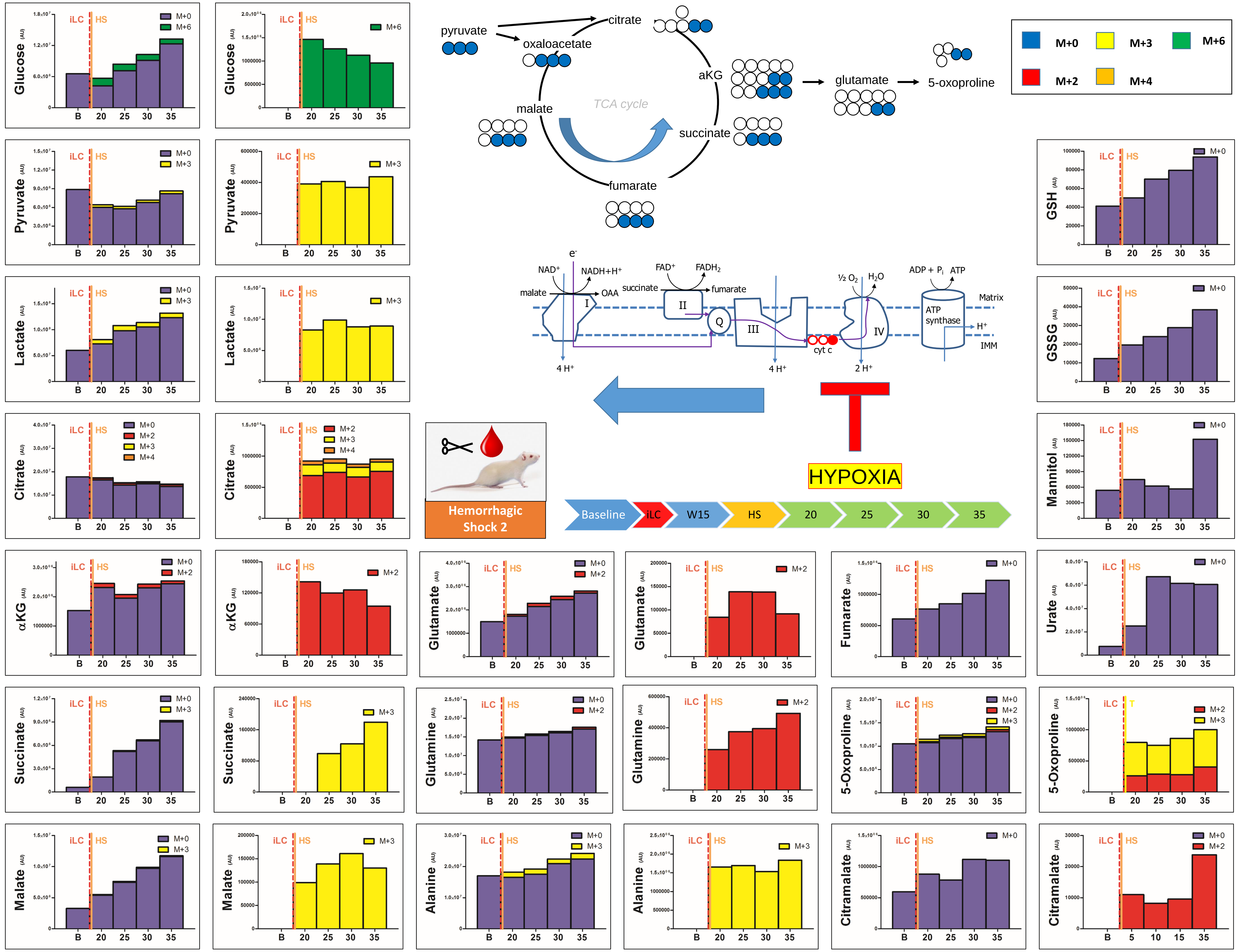

**Additional Figure 5** - Blood from trauma/hemorrhagic shock rats (laparotomy with bowel crush, with hemorrhagic shock to MAP<30) was withdrawn before injection (baseline – B) of labeled 13C-glucose (iLC). After waiting for 15 minutes (W15), hemorrhagic shock was induced to MAP<30 and blood was then collected at 20, 25, 30 and 35 minutes from iLC. Metabolites of glycolysis and Krebs cycles were monitored, as they have been previously shown to increase in plasma after trauma/hemorrhagic shock [14]. In left, the total levels of the metabolite (integrated peak areas – arbitrary units) are indicated through stacked bar graphs, including the unlabeled parent (blue – M+0) and heavy isotopologues (either M+2, M+3, M+4, M+6 depending on the expected labeling pattern from catabolism of 13C-glucose). In the right hand panels, only heavy isotopologues (red, yellow, orange, green) are shown. As soon as 25 minutes after iLC, hemorrhagic shock induced accumulation of lactate and unlabeled glucose (indicative of ongoing gluconeogenesis) and late Krebs cycle intermediates (succinate, fumarate, malate), increased levels of glutamate and totally unlabeled urate, polyamines (spermidine), glutathione (either reduced – GSH and oxidized – GSSG), mannitol and (minimally labeled) citramalate. M+3 labeling in malate and succinate is suggestive of malate generation from oxaloacetate obtained via pyruvate carboxylase activity and backwards fluxing of complex I and II to generate malate and succinate in the absence of oxygen as a final electron acceptor (following HS– top right corner). Extended version of in text Figure 6.
